# Targeting lipocalin-2 for multiple sclerosis: a dual role in diagnosis and therapy

**DOI:** 10.3389/fimmu.2026.1718318

**Published:** 2026-03-04

**Authors:** Ruqayya Afridi, Won-Ha Lee, Minsoo Song, Kyoungho Suk

**Affiliations:** 1 Department of Pharmacology, School of Medicine, Kyungpook National University, Daegu, Republic of Korea; 2 Department of Biomedical Sciences, Brain Korea 21 Four Kyungpook National University (KNU) Convergence Educational Program of Biomedical Sciences for Creative Future Talents, Kyungpook National University, Daegu, Republic of Korea; 3 School of Life Sciences, Kyungpook National University, Daegu, Republic of Korea; 4 Department of Medicinal Chemistry, New Drug Discovery Center (NDDC), Daegu Gyeongbuk Medical Innovation Foundation (K-MEDI hub), Daegu, Republic of Korea; 5 Brain Science and Engineering Institute, Kyungpook National University, Daegu, Republic of Korea

**Keywords:** biomarker, lipocalin-2, multiple sclerosis, neuroinflammation, therapeutic target

## Abstract

The discovery of novel biomarkers and therapeutic targets is essential for advancing multiple sclerosis (MS) treatment strategies. Lipocalin-2 (LCN2), a 25-kDa glycoprotein, has gained considerable attention for its diverse roles in immune regulation and neuroinflammation. Its expression varies across MS subtypes and disease stages, influencing both peripheral immune responses and central nervous system pathology. Growing evidence has demonstrated the involvement of LCN2 in modulating immune cell function, glial reactivity, and blood-brain barrier integrity. Clinical studies have consistently correlated LCN2 levels in patient biofluids with disease parameters, supporting its potential as a biomarker. Moreover, experimental studies targeting LCN2 have shown promising therapeutic potential. This review examines the role of LCN2 in MS, focusing on its impact on peripheral immune cells, neuroinflammation, and its viability as a biomarker and therapeutic target. We also discuss the relevance of LCN2-targeting therapies within the evolving MS treatment landscape, underscoring the need for further research in this area.

## 1 Introduction

Multiple sclerosis (MS) is a complex autoimmune disorder of the central nervous system (CNS), characterized by chronic inflammation, demyelination, and neurodegeneration (1). This disease primarily affects young adults, leading to significant disability and a substantial socioeconomic burden (2). Despite advances in MS diagnosis and treatment, the precise molecular mechanisms underlying its pathogenesis remain poorly understood. The heterogeneous nature of MS, involving both immune and neurodegenerative components, complicates treatment strategies and highlights the need for novel biomarkers and therapeutic targets.

In recent years, lipocalin-2 (LCN2), also known as neutrophil gelatinase-associated lipocalin (NGAL), has gained increasing attention as a potential key player in MS pathophysiology (3–6). LCN2 is a 25-kDa secreted glycoprotein belonging to the lipocalin family, which is widely known for its role in iron homeostasis and innate immunity (3, 7–9). However, it is now known to be also involved in several other biological processes, including the transport of small hydrophobic molecules, cell proliferation, apoptosis, and inflammation in both physiological and pathological conditions (3, 10, 11). Increasing evidence suggests that LCN2 is associated with MS pathology by modulating systemic inflammation, neuroinflammation, glial alterations, and blood-brain barrier (BBB) dysfunction (12).

Elevated LCN2 levels have been consistently detected in both cerebrospinal fluid (CSF) and serum of MS patients, correlating with disease severity and progression (13). Moreover, experimental studies using animal models of MS have demonstrated genetic or pharmacological inhibition of LCN2 can significantly influence disease outcomes, highlighting its potential as both a biomarker and a therapeutic target (6). This review aims to comprehensively examine the role of LCN2 in MS pathogenesis, its utility as a biomarker, and its therapeutic potential. We will explore the molecular mechanisms underlying the involvement of LCN2 in immune responses and neuroinflammation, assess its relevance as a biomarker across different MS subtypes, and discuss emerging therapeutic strategies targeting LCN2.

## 2 LCN2 and peripheral immune responses in MS

The regulation of peripheral immune responses by LCN2 plays a critical role in MS pathophysiology (14). The role of LCN2 in innate immunity, particularly in modulating the activity and polarization of macrophages, neutrophils, and T-cells, has been linked to its ability to regulate inflammatory responses and mediate immune cell crosstalk in MS pathology (5, 15). Immune dysfunction in MS is characterized by the infiltration of autoreactive T-cells and monocytes into the CNS, triggering inflammatory cascades and neuronal damage. Increasing evidence has revealed complex interactions between LCN2 and various immune cell populations, underscoring its fundamental role in both disease initiation and progression (**Table 1**).

**Table 1:** Summary of LCN2 involvement in regulating peripheral immune cell responses and neuroinflammation in MS pathogenesis.

Mechanisms	Key findings	References
Regulation of peripheral immune cell responses			
T-cell Responses	Promotes pathogenic Th1 and Th17 cell development	(5)
	Enhances MOG-specific T-cell proliferation	
	Upregulates pro-inflammatory cytokines (IL-17, IFN-γ)	
Monocyte/macrophage Function	Enhance monocyte chemotaxis	(16)
	Drives M1 pro-inflammatory macrophage polarization	
	Contributes to adipose tissue fibrosis in EAE model	
Neutrophil Activity	Significantly enriched in neutrophils in virus-induced MS models	(6, 19)
	Promotes CXCL9/CXCL10 expression facilitating T-cell recruitment	
	Forms complex with MMP-9 enhancing inflammatory response	
	Elevated in MS patient neutrophils correlating with activation markers	
Gut Inflammation	Increased in fecal samples from MS patients	(6)
	Mediates IL-17A-driven neutrophil recruitment to intestine	
	Contributes to gut dysbiosis and microbial imbalance	
Regulation of neuroinflammation		
Astrocyte Function	Primarily produced by astrocytes in CNS during inflammation	(5)
	Upregulated response to TNF-α, IL-1β, and IL-17	
	Drives astrocyte reactivity and GFAP expression	
Oligodendrocyte Effects	Impairs oligodendrocyte differentiation, proliferation, and survival	(13, 24)
	Reduces myelination in a dose-dependent manner	
	Activates SLC22A17/EGR1 signaling pathway	
Microglial Activation	Promotes pro-inflammatory microglial phenotype	(5, 27)
	Increases production of inflammatory cytokines	
BBB damage	Disrupts BBB integrity	(5, 17)
	Modulates tight junction proteins	
	Regulates matrix metalloproteinase expression	
	Facilitates peripheral immune cell infiltration	

Th1, T helper 1 cells; Th17, T helper 17 cells; MOG, myelin oligodendrocyte glycoprotein; IL-17, Interleukin 17; IFN-γ, interferon gamma; M1, Type 1 (pro-inflammatory) macrophages; EAE, experimental autoimmune encephalomyelitis; MS, multiple sclerosis; CXCL9/CXCL10, chemokine (C-X-C motif) ligands 9 and 10; MMP-9, matrix metalloproteinase 9; CNS, central nervous system; TNF-α, tumor necrosis factor alpha; IL-1β, interleukin 1 beta; GFAP, glial fibrillary acidic protein; SLC22A17, solute carrier family 22 member 17; EGR1, early growth response 1; BBB, blood-brain barrier.

The impact of LCN2 on T-cell responses is a crucial factor in MS pathogenesis (5). Elevated LCN2 levels significantly are associated with altered T-cell differentiation patterns, promoting the development of pathogenic Th1 and Th17 cells through both direct and indirect mechanisms (5). In experimental autoimmune encephalomyelitis (EAE), an animal model of MS, LCN2 enhances encephalitogenic T-cell responses by promoting MOG-specific T-cell proliferation and upregulating interleukin-17 (IL-17) and interferon-γ (IFN-γ) through transcription factors such as *Rorc* (encoding RORγt) and *Tbet* (encoding T-bet) (5). This amplification of Th1 and Th17 immune responses contributes to disease progression. Genetic ablation of *Lcn2* reduces inflammatory infiltration, mitigates neuroinflammation, and limits demyelination, further emphasizing its pathogenic role in both CNS and peripheral immunity. Beyond its role in T-cell activation, LCN2 also enhances antigen presentation by innate immune cells, further shaping T-cell responses in a broader immunological context.

The influence of LCN2 on myeloid cell populations, particularly monocytes, dendritic cells, and neutrophils, represents another key aspect of its immune regulatory function in MS. LCN2 significantly enhances monocyte chemotaxis and drives their differentiation toward pro-inflammatory M1 macrophage phenotypes (16). In EAE, dysfunctional adipocytes release LCN2 through a redox-dependent mechanism. This promotes M1-like macrophage infiltration and adipose tissue fibrosis, features consistent with a cachectic phenotype (16). Adipose-specific *Lcn2* deficiency reduces weight loss and inflammatory macrophage infiltration in the spinal cord, while knockdown of *Lcn2* expression limits the lipolytic response to inflammation. These findings suggest the role of LCN2 in linking adipose dysfunction to neuroinflammation, thereby contributing to MS pathogenesis. Activated macrophages further exacerbate BBB disruption and CNS inflammation. Likewise, LCN2 influences dendritic cell maturation and antigen-presenting capabilities, significantly impacting T-cell priming and activation. This creates a feed-forward loop that perpetuates inflammatory responses, reinforcing its role in MS progression.

Given its historical identification as neutrophil gelatinase-associated lipocalin, the effects of LCN2 on neutrophil function warrant particular attention. The LCN2 protein regulates multiple aspects of neutrophil behavior, including their recruitment to inflammatory sites, survival, activation, and specialized functions such as degranulation and neutrophil extracellular trap (NET) formation (6). Notably, LCN2 expression was significantly enriched in neutrophils from a virus-induced animal model of MS (17). The subsequent upregulation of LCN2 was associated with increased expression of chemokines CXCL9 and CXCL10, facilitating T-cell recruitment and worsening T-cell-mediated demyelination. Furthermore, LCN2 formed a complex with matrix metalloproteinase (MMP-9), stabilizing MMP-9 against degradation and enhancing its enzymatic activity, thereby contributing to the heightened inflammatory response and demyelination observed in this study (17). In the EAE model, LCN2 expression is significantly elevated in secondary lymphoid organs, where neutrophils serve as a key source of LCN2, while dendritic cells express the LCN2 receptor 24p3R, suggesting a role for LCN2-24p3R interactions in antigen presentation and T-cell activation (5).

Neutrophils also play a critical role in the gut inflammation observed in MS-associated dysbiosis (6). In EAE mice, neutrophil infiltration into the large intestine was significantly increased, coinciding with elevated levels of fecal LCN2, which acts as a biomarker of intestinal inflammation. Neutrophil recruitment was mediated by IL-17A signaling, prominently produced by gut-infiltrating Th17 cells. Once in the intestinal mucosa, neutrophils contribute to dysbiosis by releasing antimicrobial proteins such as neutrophil elastase and reactive oxygen species, which disrupt microbial homeostasis (18). These findings suggest that neutrophil infiltration may contribute to gut inflammation and microbial imbalance in MS, potentially linking intestinal immune dysregulation to CNS autoimmunity.

Proteomic analysis revealed a significant increase in LCN2 abundance within neutrophils from MS patients compared to healthy controls, suggesting an enhanced inflammatory state in MS (19). Levels of LCN2, a key component of neutrophil secondary granules, were elevated in MS neutrophils. This was correlated with increased activation markers, including CD11b and CD66b, indicating increased inflammatory activity (19). Furthermore, LCN2 contributes to neutrophil-driven dysregulation of adaptive immunity. When neutrophils derived from MS patients were co-cultured with T-cells, these neutrophils failed to restrict the inflammatory activation of T-cells, whereas neutrophils obtained from healthy donors were able to control T-cell activation (19). These findings highlight LCN2 as a potential biomarker of neutrophil dysfunction in MS and a contributor to disease-associated immune dysregulation.

**Figure 1:**
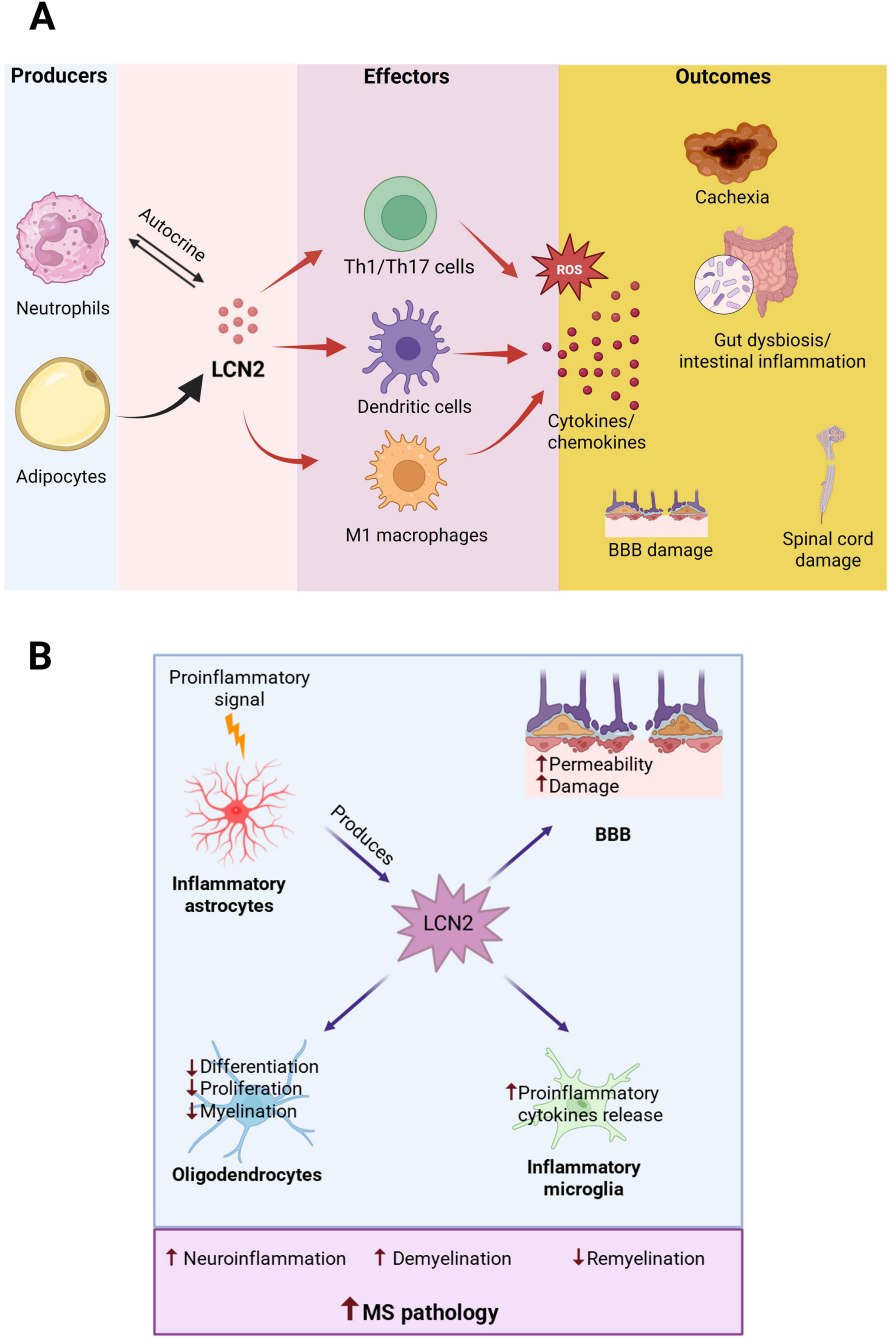
LCN2-mediated inflammatory pathways linking peripheral immunity and CNS pathology in MS. **(A)** Peripheral actions of LCN2. Neutrophils and adipocytes are major peripheral sources of LCN2, with neutrophils also engaging in autocrine signaling. Peripheral LCN2 promotes activation of Th1/Th17 cells, dendritic cells, and M1 macrophages, leading to increased reactive oxygen species (ROS) production and release of pro-inflammatory cytokines and chemokines. These inflammatory cascades contribute to cachexia, gut dysbiosis, and intestinal inflammation, which collectively amplify peripheral immune activation and exacerbate blood-brain barrier (BBB) disruption and spinal cord damage. **(B)** Central actions of LCN2. In the CNS, inflammatory astrocytes produce LCN2 in response to pro-inflammatory signals. Astrocyte-derived LCN2 increases BBB permeability, suppresses oligodendrocyte differentiation, proliferation, and myelination, and enhances pro-inflammatory cytokine release from activated microglia. Together, these processes drive neuroinflammation, promote demyelination, and impair remyelination, thereby exacerbating MS pathology. Arrows indicate direction of effect; ↑ denotes increased activity and ↓ denotes inhibition.

Changes in peripheral LCN2 levels may serve as valuable indicators of immune activation and disease progression (**Figure 1**). Additionally, the broad influence of LCN2 across multiple immune cell populations suggests that targeting LCN2 or its signaling could offer a comprehensive approach to MS treatment, potentially addressing multiple pathogenic mechanisms simultaneously.

## 3 LCN2 and neuroinflammation in MS

The central role of LCN2 in neuroinflammation is a critical aspect of MS pathophysiology within the CNS. LCN2 expression is markedly upregulated during neuroinflammatory episodes, with astrocytes serving as the primary source of this protein in the CNS (**Figure 1**). This increased expression closely correlates with disease progression and severity in both MS patients and EAE models. Astrocytes are the primary cellular source of LCN2 during neuroinflammatory conditions, with production increasing significantly in response to pro-inflammatory mediators such as tumor necrosis factor-α (TNF-α), IL-1β, and IL-17 (20). Astrocyte-derived LCN2 has been shown to be involved in a self-perpetuating inflammatory cycle through autocrine signaling, driving continued astrocyte reactivity, which is characterized by morphological changes and increased expression of glial fibrillary acidic protein (GFAP) (3). The resulting reactive astrocytes further amplify the inflammatory cascade by releasing additional pro-inflammatory mediators.

The contribution of LCN2 in augmenting neuroinflammation in MS pathophysiology has been demonstrated in multiple experimental models (**Table 1**) (14). In EAE mice, LCN2 expression significantly increases in astrocytes during disease onset, leading to elevated production of chemotactic molecules, including CXCL10 and CCL2 (5). These chemokines facilitate the infiltration of peripheral immune cells across the BBB, exacerbating neuroinflammation (5). The pathological significance of these mechanisms (5), who reported that *Lcn2* gene knockout (KO) EAE mice exhibited reduced clinical severity scores and attenuated neuroinflammatory responses compared to wild-type (WT) mice. Supporting these findings, another study demonstrated reduced neuroinflammation in *Lcn2* KO EAE mice, with notable decreases in demyelination, despite similar overall clinical scores between KO and WT mice (21). This selective improvement in myelin preservation further underscores the specific role of LCN2 in MS-related pathology. Additionally, in the EAE model, LCN2 was among the most differentially expressed genes in the motor and sensory cortex (22). In the cuprizone demyelination model, increased astrocytic LCN2 was consistently identified as a marker of inflammatory astrocytes across multiple brain regions, including the corpus callosum, hippocampus, and cortex (23).

The detrimental effects of LCN2 on remyelination processes are linked to its effects on oligodendrocyte biology. LCN2 negatively influences oligodendrocyte differentiation, proliferation, survival, and function (13, 24). Additionally, LCN2 upregulates the expression of TNF-α and MMP-9, which directly impair oligodendrocyte myelination capacity (5). In neuron-glia co-culture experiments, recombinant LCN2 treatment reduced myelination in a dose-dependent manner (13). Furthermore, LCN2 knockdown in the cuprizone model preserved white matter integrity in the corpus callosum and facilitated remyelination during the recovery phase, a positive outcome not observed in WT animals (24). Mechanistically, LCN2 impairs oligodendrocyte progenitor cell differentiation by activating the SLC22A17/early growth response protein-1 signaling pathway (24).

However, the literature presents some conflicting findings regarding LCN2’s role in oligodendrocyte regulation (24, 25). Studies using astrocyte-specific *Lcn2* deletion have shown reduced levels of both mature and progenitor oligodendrocytes in EAE models despite an overall improvement in clinical scores in these conditional knockout mice compared to WT animals (12). Additionally, in a combined cuprizone + EAE model, *Lcn2* KO mice exhibited paradoxically increased neuroinflammation and demyelination (25). These seemingly contradictory results likely reflect the time-dependent effects of LCN2 on myelination, as the protein primarily impacts actively myelinating oligodendrocytes and has minimal effect once myelination is completed (13).

In MS and EAE, LCN2 expression is consistently increased within affected CNS regions; however, its functional role appears to vary depending on disease context and experimental conditions (5, 26). Divergent outcomes reported in EAE likely reflect differences in the timing of analysis, as LCN2 may exert distinct effects during acute inflammatory phases compared to later, chronic stages of disease (5, 26). In addition, EAE outcomes are sensitive to variations in induction protocols, disease severity, and the CNS region analyzed, all of which can influence the apparent contribution of LCN2 to lesion formation.

Importantly, differences between disease models further shape LCN2-associated effects. While EAE predominantly models autoimmune-driven inflammation of the spinal cord, the cuprizone model induces primary oligodendrocyte loss and brain-intrinsic demyelination in the absence of peripheral immune cell infiltration, thereby revealing complementary and context-specific functions of LCN2 (25). The time-dependent effects of LCN2 on myelination occur because the protein primarily impacts actively myelinating oligodendrocytes and has minimal effect once myelination is completed (13). Together with the cell-type-specific expression of LCN2*—*restricted to distinct subpopulations of reactive astrocytes and infiltrating myeloid cells*—*these factors likely underlie the variable and sometimes opposing effects attributed to LCN2 across studies (5, 13, 26).

LCN2-induced neuroinflammation in MS pathology also encompasses microglial activation, increased infiltration of peripheral immune cells, and BBB deficits (16). The impact of LCN2 on microglial function is another critical aspect of neuroinflammation in MS. Microglia respond to elevated LCN2 levels by adopting a pro-inflammatory phenotype, marked by enhanced production of inflammatory cytokines and chemokines (27). *In vitro* treatment of microglial cells with LCN2 increases the release of various pro-inflammatory cytokines, including TNF-α. This activation state promotes further recruitment of peripheral immune cells into the CNS, contributing to the establishment of a chronic inflammatory environment. The interaction between LCN2 and microglia appears to be bidirectional, as activated microglia can also produce LCN2, albeit at lower levels than astrocytes (5). In *Lcn2* KO EAE mice, the inflammatory activation of microglia was reduced, leading to less MS-related disease pathology (5).

BBB integrity is significantly influenced by LCN2 during MS progression. This protein contributes to BBB disruption through multiple mechanisms, including modulation of tight junction proteins and regulation of matrix metalloproteinase expression (5). This breakdown of BBB integrity facilitates the infiltration of peripheral immune cells into the CNS, further exacerbating the inflammatory response. Additionally, the role of LCN2 in iron trafficking across the BBB adds another layer of complexity to its involvement in neuroinflammation. In *Lcn2* KO EAE mice, the levels of pro-inflammatory cytokines in the cerebellum were lower compared to WT EAE mice, while anti-inflammatory cytokines were upregulated (21).

## 4 LCN2 as a biomarker and therapeutic target for MS

The potential of LCN2 as both a biomarker and therapeutic target in MS has garnered significant attention through clinical and preclinical investigations (26, 28). Accumulating evidence from human studies has revealed distinctive patterns of LCN2 expression across different MS subtypes, disease stages, and body fluids, highlighting its potential as a diagnostic and prognostic biomarker (**Table 2**) (26).

**Table 2:** Potential of LCN2 as a biomarker for MS.

Analyte/disease stage	Findings	References
CSF levels	Consistently elevated in MS patients compared to controls	(26)
	Particularly high during acute RRMS relapses	
	Correlates with disease progression and treatment response	
Serum levels	Increased during active disease phases	(29, 32)
	Correlates with MRI markers of disease activity	
	May predict CIS to MS transition	
	Associated with iron accumulation in the basal ganglia	
MS subtype patterns	RRMS: Fluctuating CSF levels corresponding with disease activity	(13, 30)
	Progressive MS: Sustained elevation in brain tissue/interstitial fluid	
	Changing serum levels may indicate conversion from RRMS to SPMS	

CSF, cerebrospinal fluid; RRMS, relapsing-remitting multiple sclerosis; CIS, clinically isolated syndrome; MRI, magnetic resonance imaging; SPMS, secondary progressive multiple sclerosis.

Clinical evidence consistently demonstrates elevated LCN2 levels in the CSF of MS patients compared to healthy controls and individuals with other neurological disorders (29). The potential of LCN2 as a clinically relevant biomarker in MS has been supported by accumulating evidence from both cross-sectional and longitudinal human studies. Depending on disease stage, MS subtype, and biofluid analyzed, LCN2 may function as a diagnostic, prognostic, and disease-monitoring biomarker. Distinct expression patterns of LCN2 have been reported in CSF, serum, and fecal samples (6, 29, 30), reflecting both central and peripheral inflammatory processes associated with MS pathology.

LCN2 has emerged as a marker of disease activity in multiple sclerosis, particularly in relapsing-remitting MS (RRMS). Elevated LCN2 levels are most prominent during acute relapses, underscoring its potential as a biomarker of active neuroinflammation (31). Longitudinal analyses of CSF LCN2 concentrations further demonstrate significant correlations with disease progression and treatment response, supporting its value as a monitoring biomarker for therapeutic efficacy (13).

While CSF measurements provide robust biomarker data, serum LCN2 determinations offer a less invasive alternative, albeit with more variable results (32). Several studies have reported increased serum LCN2 concentrations in MS patients, particularly during periods of active disease. Importantly, serum LCN2 levels correlate significantly with MRI markers of disease activity, reinforcing their potential utility for disease monitoring. Reduced circulating miRNA-484 levels have also been associated with increased LCN2 concentrations, correlating with greater disability in MS patients (31).

In addition to its role in disease monitoring, LCN2 shows promise as a diagnostic biomarker. Elevated serum and CSF LCN2 levels have been shown to predict conversion from clinically isolated syndrome (CIS) to clinically definite MS, supporting its utility in early disease identification (29). Furthermore, CSF LCN2 levels strongly correlate with MRI-detected iron accumulation in the basal ganglia, linking LCN2 to early pathological changes relevant to diagnosis (29).

Distinct patterns of LCN2 expression have been observed across MS subtypes. Progressive MS, including secondary progressive MS (SPMS) and primary progressive MS (PPMS), typically exhibit sustained elevations of LCN2 in CSF, whereas RRMS patients show fluctuating LCN2 levels corresponding to inflammatory disease activity (13). These subtype-specific expression profiles likely reflect differing pathological mechanisms and may improve disease classification and prognostic prediction.

Genetic studies have identified single nucleotide polymorphisms in STK11, encoding Liver Kinase-B1 (LKB1), as MS risk factors, with LCN2 playing a central role in this association (30). African American MS patients carrying STK11 polymorphisms exhibit higher LCN2 levels in both CSF and serum, with concentrations declining as the disease progresses. Notably, changes in LCN2 levels may signal transition from RRMS to SPMS, suggesting that elevated LCN2 in earlier disease stages are associated with pathological progression.

Beyond its neurological relevance, LCN2 also serves as a biomarker of MS-associated systemic complications, particularly gut dysbiosis. RRMS patients consistently exhibit elevated fecal LCN2 levels, alongside increased markers of intestinal inflammation (6). Building on these findings, a recent clinical trial utilized fecal LCN2 as an endpoint to evaluate the efficacy of high-fiber dietary interventions aimed at correcting gut dysbiosis in MS patients (33).

The therapeutic targeting of LCN2 has been actively investigated in preclinical models, particularly in the EAE animal model of MS. Studies using *Lcn2*-deficient mice have shown reduced disease severity and altered immune responses, suggesting that LCN2 inhibition could have therapeutic potential (5, 16). Experimental interventions have demonstrated multiple mechanisms by which LCN2-targeted therapies may be beneficial in MS-related pathology (5, 24), including enhanced BBB integrity, decreased inflammatory cell infiltration, altered T-cell responses, and changes in astrocyte reactivity (6, 16). These broad effects on both peripheral immunity and CNS inflammation suggest that targeting LCN2 could provide advantages over existing therapies that focus primarily on immune suppression (16).

Despite significant advances in MS treatment, major challenges remain, particularly in addressing disease progression and neurodegeneration (34). Current therapies primarily target peripheral immune cells to prevent their infiltration into the CNS, while their effects on resident brain cells remain less understood (34). First-line treatments, such as interferon-beta and glatiramer acetate, reduce relapse rates to a moderate degree but have limited effects on long-term disability (35). Second-line therapies, including natalizumab, alemtuzumab, and ocrelizumab, offer greater efficacy but are associated with serious risks, such as progressive multifocal leukoencephalopathy and secondary autoimmune disorders (36). Newer agents, including sphingosine-1-phosphate receptor modulators, dimethyl fumarate, and anti-CD20 antibodies, have shown promise but remain largely focused on immune modulation rather than direct neuroprotection (36).

Natalizumab, a treatment for highly active RRMS, primarily functions by inhibiting leukocyte chemotaxis. It does so by blocking alpha-4 integrin receptors (α4-subunit of α4β1 and α4β7 integrins), preventing their interaction with vascular cell adhesion molecule-1 (VCAM-1) on endothelial cells (37). Notably, in EAE mice models, natalizumab administration reduced astrocytic LCN2 expression during the disease onset phase and lowered CSF LCN2 levels, suggesting a potential mechanistic link between current MS therapies and LCN2 regulation (28). Given the demonstrated roles of LCN2 in both inflammation and neurodegeneration, further exploration of its therapeutic potential could offer novel strategies for addressing MS pathogenesis. As research progresses, LCN2-targeting approaches may provide a means to address multiple disease mechanisms simultaneously, filling critical gaps in current treatment options and improving patient outcomes.

Despite compelling preclinical evidence, the translational targeting of LCN2 in humans presents important challenges that must be carefully considered. LCN2 plays essential physiological roles in iron homeostasis, antimicrobial defense, and tissue protection, particularly during acute infection and systemic inflammation (8). Consequently, complete or chronic systemic inhibition of LCN2 may carry risks, including increased susceptibility to infections or dysregulated iron metabolism (35, 36, 38). These considerations argue against indiscriminate LCN2 blockade as a therapeutic strategy in MS.

While preclinical studies support the pathogenic involvement of LCN2 in MS-related inflammation and demyelination, translation to human therapy presents significant challenges rooted in essential physiological roles of LCN2. LCN2-deficient mice demonstrate impaired bacterial clearance in infection models, and disruption of iron regulation could lead to additional complications in patients with chronic inflammatory disease (35, 36, 39). From a translational perspective, more feasible approaches may involve partial modulation of LCN2 signaling, temporal restriction to active inflammatory phases, or cell-type-specific targeting. Advances in CNS-targeted delivery systems, antibody engineering, and RNA-based therapeutics may eventually enable selective modulation of pathogenic LCN2 signaling while preserving its systemic protective functions.

Importantly, biomarker-guided patient stratification may further enhance therapeutic feasibility by identifying MS subgroups with elevated or persistent LCN2 expression who are most likely to benefit from LCN2-directed interventions (30). In this context, LCN2-targeted strategies are best envisioned as adjunctive or precision therapies, complementing existing disease-modifying treatments rather than replacing them. Together, these considerations underscore both the promise and the complexity of translating LCN2 biology into safe and effective therapeutic approaches for MS.

## 5 Conclusion and future perspectives

LCN2 stands out as a unique biomarker due to its subtype-specific expression and correlation with disease activity, offering potential applications in diagnosis, disease monitoring, and treatment response assessment in MS. Its detectability across multiple biofluids (CSF, serum, and feces) enhances its clinical utility. In addition to its diagnostic potential, LCN2 plays a multifaceted role in MS pathogenesis, influencing T-cell responses, neutrophil mobilization, macrophage polarization, blood-brain barrier integrity, and glial reactivity. This broad involvement suggests that LCN2-targeted therapies have the potential to influence multiple disease mechanisms, overcoming the limitations of current treatments that primarily focus on peripheral immune suppression while neglecting CNS pathology.

A growing body of evidence has revealed the significance of LCN2 in MS, highlighting its dual involvement in peripheral immune dysregulation and central neuroinflammation. Elevated LCN2 levels in patient biofluids are correlated with disease severity, while experimental models demonstrate the therapeutic benefits of LCN2 inhibition. These findings support LCN2 as both a promising biomarker and a potential therapeutic target. Future research should focus on translating LCN2 biology into clinical utility by prioritizing cell-type-specific targeting strategies to inhibit pathogenic astrocytic LCN2 while preserving beneficial functions. Standardized longitudinal human studies across MS subtypes are needed to validate LCN2 as a biomarker for disease progression and treatment response. Integrating multi-omics data with advanced imaging may enable patient stratification and personalized LCN2-targeted therapies. Mechanistic studies clarifying when LCN2 is protective versus harmful will be essential to define optimal therapeutic windows. Finally, clinical trials should stratify patients by LCN2 profiles, assess combination therapies, and ensure long-term safety given the roles of LCN2 in iron homeostasis and host defense.
